# Social Freezing: Pressing Pause on Fertility

**DOI:** 10.3390/ijerph18158088

**Published:** 2021-07-30

**Authors:** Valentin Nicolae Varlas, Roxana Georgiana Bors, Dragos Albu, Ovidiu Nicolae Penes, Bogdana Adriana Nasui, Claudia Mehedintu, Anca Lucia Pop

**Affiliations:** 1 Department of Obstetrics and Gynaecology, Filantropia Clinical Hospital, 011171 Bucharest, Romania; valentin.varlas@umfcd.ro (V.N.V.); roxana-georgiana.bors@rez.umfcd.ro (R.G.B.); dragos.albu@umfcd.ro (D.A.); 2 Department of Obstetrics and Gynaecology, “Carol Davila” University of Medicine and Pharmacy, 37 Dionisie Lupu St., 020021 Bucharest, Romania; 3 Department of Intensive Care, University Clinical Hospital, “Carol Davila” University of Medicine and Pharmacy, 37 Dionisie Lupu St., 020021 Bucharest, Romania; 4 Department of Community Health, “Iuliu Hațieganu” University of Medicine and Pharmacy, 6 Louis Pasteur Street, 400349 Cluj-Napoca, Romania; 5 Department of Obstetrics and Gynaecology, Nicolae Malaxa Clinical Hospital, 020346 Bucharest, Romania; claudia.mehedintu@umfcd.ro; 6 Department of Clinical Laboratory, Food Safety, “Carol Davila” University of Medicine and Pharmacy, 6 Traian Vuia Street, 020945 Bucharest, Romania; anca.pop@umfcd.ro

**Keywords:** social egg freezing, elective egg freezing, oocyte cryopreservation, fertility preservation, age-related infertility, oocyte vitrification, delayed childbearing

## Abstract

Increasing numbers of women are undergoing oocyte or tissue cryopreservation for medical or social reasons to increase their chances of having genetic children. Social egg freezing (SEF) allows women to preserve their fertility in anticipation of age-related fertility decline and ineffective fertility treatments at older ages. The purpose of this study was to summarize recent findings focusing on the challenges of elective egg freezing. We performed a systematic literature review on social egg freezing published during the last ten years. From the systematically screened literature, we identified and analyzed five main topics of interest during the last decade: (a) different fertility preservation techniques, (b) safety of freezing, (c) usage rate of frozen oocytes, (d) ethical considerations, and (e) cost-effectiveness of SEF. Fertility can be preserved for non-medical reasons through oocyte, embryos, or ovarian tissue cryopreservation, with oocyte vitrification being a new and optimal approach. Elective oocyte cryopreservation is better accepted, supports social gender equality, and enhances women’s reproductive autonomy. Despite controversies, planned oocyte cryopreservation appears as a chosen strategy against age-related infertility and may allow women to feel that they are more socially, psychologically, and financially stable before motherhood.

## 1. Introduction

The fertility preservation field has developed over the last two decades, but data regarding its results are limited. An increasing number of women choose to delay the conception of a child for various social reasons.

Social egg freezing (SEF) allows women to preserve their fertility in anticipation of age-related fertility decline and ineffective fertility treatments at older ages. “Social freezing” is the term used when eggs or ovarian tissue are frozen for non-medical causes and used later in life. The terminology used is controversial. “Elective egg freezing” is the term preferred by most women. “Social egg freezing” highlights the fact that women’s reproductive choices are socially embedded. We often encounter the term “non-medical egg freezing” or “egg freezing for non-medical reasons”. The decision to cryopreserve oocytes to protect women against age-related fertility decline should be considered a preventive medical treatment, which led to the use of the term “AGE banking” (oocyte banking for anticipated gamete exhaustion) [1,2].

After many years of research, we have started to understand what motivates increasing numbers of women worldwide to freeze eggs for elective rather than medical reasons. They undergo this procedure for professional, personal, financial, and/or psychological reasons. The most common cause of delaying childbearing cited by women is the lack of a partner suitable for creating a family. The second reason is professional, related to completing education, career advancement, and inflexibility in the workplace, with women considering becoming pregnant before 35 years as affecting their career [1,3,4].

Elective egg freezing is allowed for healthy women age between 30 to 41 years as a solution against age-related infertility problems; it is considered to be an act of preventive medicine. In addition, this technology offers women across reproductive age the possibility of having genetic children when they reach financial stability and have sufficient maturity and emotional support [5,6].

Epidemiological studies showed that women who choose elective egg freezing are commonly Caucasian, between 36 and 40 years of age, with higher education, professional employment, and without a partner [1,3].

The reproductive window is narrower in women than in men. After the mid-thirties, women’s fertility potential decreases gradually, with lower fertility after 35 years. Women’s fertility continues to decline every year until menopause because the number and quality of the primordial follicles of oocytes decrease, associated with lower chances of the oocytes being fertilized, higher risk of abnormal embryos, and fetal loss [1,3,7].

Women who choose elective oocyte cryopreservation may address their gynecologist or fertility preservation team, consisting of an embryologist, fertility specialist, and a psychologist or counselor [8]. However, for quality decision-making, they should be informed of the risks of the procedure, benefits and costs, the success rates, long-term outcomes regarding physical health, psychological well-being, currently known data on the health of children born from frozen oocytes, duration of storage of frozen eggs, and sign an informed consent form [2,5,9].

From a financial perspective, it is preferable to preventively cryopreserve eggs at a younger age than pay for possibly unsuccessful fertility treatments due to older age [5,10].

**Figure 1:**
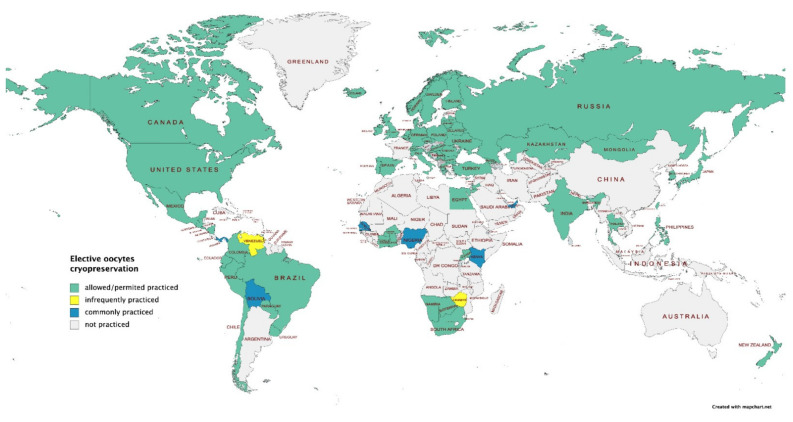
Elective oocytes cryopreservation: worldwide map (data provided by International Federation of Fertility Societies’ Surveillance [11], created with Mapchart.net).

Since 2012, increasing numbers of fertility centers worldwide have been providing elective oocyte cryopreservation to women who want to maintain their reproductive potential long-term [6] (Figure 1). In addition, an increasing number of women choose to delay the conception of a child for social reasons; yet, limited data are available on the topic.

The purpose of our study was to systematically review the recent findings focusing on the elective egg freezing challenges published in the last decade in primary databases.

## 2. Materials and Methods

We performed a systematic qualitative review in the present study according to the Preferred Reporting Items for Systematic Review and Meta-Analysis (PRISMA) guidelines. For this purpose, we performed a preliminary search on Google Scholar. We systematically searched PubMed^®^/MEDLINE (http://www.ncbi.nlm.nih.gov/PubMed, accessed on 15 January 2021) and Web of Science^®^ databases for recent literature as systematic reviews (SRs), meta-analyses (MAs), (randomized) clinical trials (RCT/CT), and clinical case series (CCS) related to elective egg freezing.

We restricted searching for articles written in English for the prior ten-year period using the specific keywords “social egg freezing”, OR “elective egg freezing”, OR “oocyte cryopreservation”, OR “fertility preservation”, OR “age-related infertility” AND “Romania”. We selected recent eligible studies after we screened titles, abstracts, and full-text articles.

For data extraction, the following data were selected: author(s), year of publication, study country, the aim of the study, study design, and main results. We selected CTs and CCS as presented below for all the data searched and retrieved from the database sources. Two independent investigators extracted the data and selected a sample of eligible studies, achieving good agreement. Firstly, we screened articles by title and abstract, and then by full text, with snowball searches of key papers. Finally, we excluded duplicates and articles not fulfilling the search criteria.

Data analysis was performed by three authors (R.G.B., A.L.P., and B.A.N.). Over 3000 studies with or without a control group were identified and screened for eligibility. According to the topic search, the data extracted included demographic variables, number of participants in the study, treatment, side effects profile, and associated comorbidities. We completed the data collection in February 2021. The quality of the studies selected for review was evaluated. Thirty-seven references were included in the present review, centered on the five main topics included in the search.

The statistical analysis was performed using Microsoft Excel^®^ in 2013 (Microsoft^®^ Corporation, Redmond, WA, USA).

## 3. Results

### 3.1. Study Selection

#### 3.1.1. PubMed^®^/MEDLINE Search

**Figure 2:**
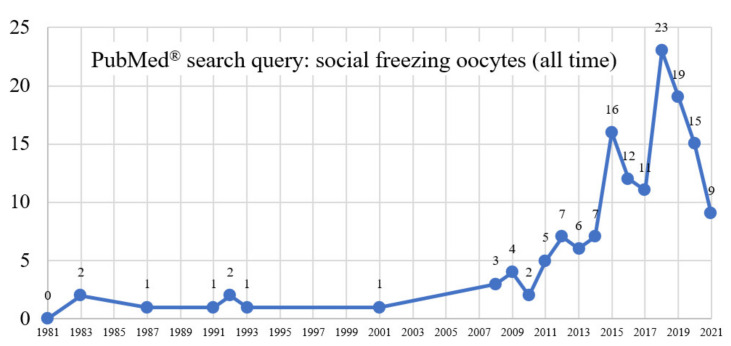
A systematic search for the keywords “social freezing (oocytes)” on the PubMed^®^ database (all-time topic) retrieved 124 results (30 reviews and meta-analysis), filtered to 54 papers refined to Medline and 25 refined to “human” since the first clinical report in 1983 [12], one systematic review [13], with three papers published during the last year.

“Elective” AND “egg” AND “freezing” (reviews and clinical trials) retrieved 78 combined results, “oocyte cryopreservation” (human) retrieved 3593 studies (all-time, since 1983) with 108 RCTs, of which 54 were performed during the past ten years and 4 RCTs during the last year. The search refined to Romania retrieved four results; no research was conducted in Romania during the study period (Figure 2).

#### 3.1.2. Web of Science^®^ Database Search

For this search, the search term TITLE was (social) AND TOPIC was (egg freezing) The timespan searched was all years. We searched the following databases: WOS, BCI, CCC, DRCI, DIIDW, KJD, Medline^®^, RSCI, Scielo, and Zoorec. The search language auto-retrieved 73 results: 69 in the Web of Science Core Collection and 46 in Medline^®,^ with 15 articles and 12 reviews. After the two databases were searched, we selected 47 clinical trials and 11 reviews as eligible, with 37 articles included in the present study, centered on five main topics: (1) fertility preservation techniques, (2) safety of freezing, (3) usage rate of the frozen oocytes, (4) ethical considerations, and (5) cost-effectiveness of SEF. 

**Figure 3:**
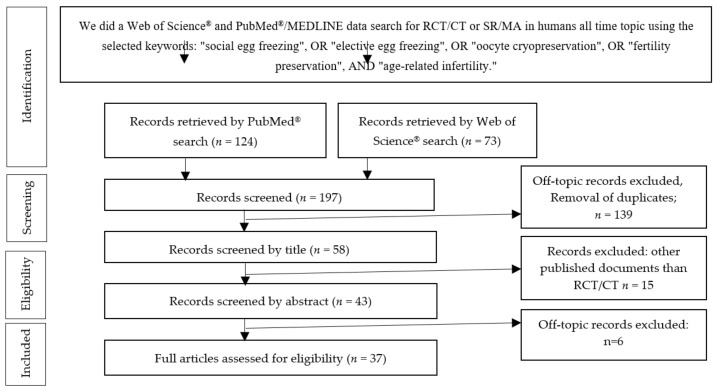
Preferred Reporting Items for Systematic Review and Meta-Analysis (PRISMA) diagram describing our systematic search and study selection process (RCT, randomized clinical trial; CT, clinical trial).

More detailed information regarding the selection process is presented in the PRISMA flow diagram in Figure 3.

### 3.2. Fertility Preservation Techniques

Fertility can be preserved for medical or non-medical reasons with similar success rates [14] through oocyte, embryos, or ovarian tissue cryopreservation. Cryopreservation involves storing cells and tissues at negative temperatures for extended periods, using cryoprotective additives to prevent ice formation [15].

#### 3.2.1. Oocyte Cryopreservation

Oocyte cryopreservation is an elective method for fertility preservation for age-related infertility and involves ovarian hormonal stimulation, oocyte retrieval, freezing, and oocyte storage [3,16]. Our systematic search on the social freezing topic refined to oocyte cryopreservation refined to articles published during the last ten years ((TS = (oocyte cryopreservation)) AND TS = (techniques) AND TS = (social freezing)) retrieved 17 results.

As of 2013, the American Society for Reproductive Medicine (ASRM) no longer considered oocyte cryopreservation an experimental procedure to become a realistic option for fertility preservation against age-related decline [3].

In 2016, Argyle et al. conducted a systematic search on the topic, reporting a rise in the success rate of oocyte preservation and increased use of the vitrification technique, generating in vitro fertilisation (IVF) pregnancy rates similar to those achieved using fresh oocytes. They emphasized that vitrification is the cryopreservation technique of choice and needs a long-term follow-up of outcomes and children born from frozen-thawed oocytes [17].

As one in six Australian women and couples experience infertility, in 2018, the Australasian CREI Consensus Expert Panel on Trial evidence group (ANZSREI ACCEPT) emphasized that the technique improves cumulative live birth outcomes for women, expanding future family building options. ANZSREI produced a consensus statement on elective cryopreservation to facilitate an optimal approach for providing care and recommended the shift of terms from the potentially stigmatizing term social egg freezing to elective or planned oocyte cryopreservation [18].

Ovarian stimulation protocols can be initiated at any moment in the menstrual cycle, Random start, or DuoStim when two stimulations are achieved. Two oocytes are retrieved in the same menstrual cycle [19] to optimize the maturity, quality, and quality and quality of the oocytes [20]. In addition, preimplantation genetic tests for aneuploidy (PGT-A) revealed that ovarian stimulation does not raise the embryo aneuploidy rate [21,22].

Oocyte vitrification is the technique of choice for oocyte cryoprecipitation [17] using the Cryotop protocol employed for oocyte vitrification initially established by Kuwayama et al. in 2005 [23].

In 2021, a study published on cohort survey on 133 women who underwent social oocyte cryopreservation (OC) investigated (1) initial motivation for freezing, (2) intentions to use the oocytes, (3) intervention feedback, and (4) awareness of the success of the entire procedure for future pregnancies. The study found an average age of 38.5 years for oocyte freezing, with 55 cryopreserved oocytes; the major reported motivation is the absence of a male partner in 40% and anticipated age-related fertility decline in 42% of the respondents. The success rate of OC was overestimated in 72% of the participants, suggesting that despite prior comprehensive, personalized counseling, a realistic understanding of reproductive aging is deficient in potential patients, leading to an eventual false sense of security [24,25]. However, in previous studies, only 6% of participants in OC used their stored oocytes (due to single parenting concern) and 3% gave birth as a result [26]. Wafi et al., in 2020, revealed a significant increase in the usage rate of OC at 20.3% [27].

#### 3.2.2. Embryo Cryopreservation

**Figure 4:**
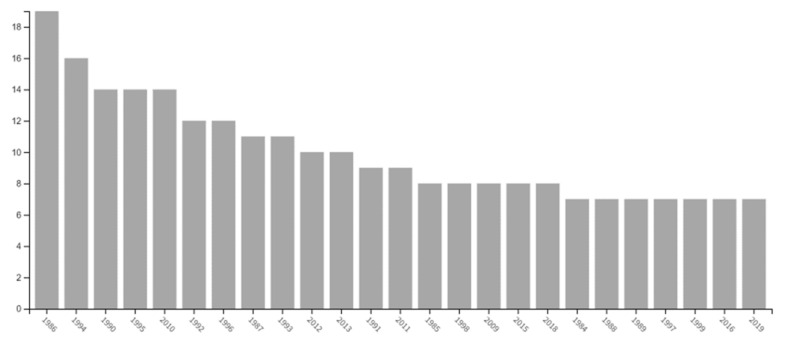
Results retrieved for on-topic search keywords “embryo” AND” cryopreservation” AND “social” AND “human” in the Web of Science^®^ Core Collection and Medline^®^ databases. The systematic search retrieved 303 results (all-time) with 90 published papers during the last ten years in all databases, from which we excluded books and others; 56 results were retrieved after data filtering, of which 23 were reviews, and 31 were articles. We identified four main papers focused on the EC topic.

Embryo cryopreservation is widely available in fertility preservation but needs legal ownership between both partners, leading to further difficulties. For example, a woman who separates from their partner with the embryos may not become pregnant if they retract their consent [2]. In addition, the complex approach toward a gamete instead of an oocyte generates a decreased interest in the topic, as shown in the retrieved database search (Figure 4).

After IVF interventions, the unused embryos are frozen and stored for further use [28], raising the issue of surplus abandoned embryos [29]. Assisted fertility procedures considering the personal and social factors to freeze embryos were studied in 2015. Goswami et al. found that couples experienced confusion associated with the term “freezing”, had concerns about the safety of the procedure, and ethical conflicts about freezing “babies” [28]. The way the couple visualizes the embryo (as a living entity, a baby, or as tissue) defines their future decision-making process.

Another implication of gamete or EC and surrogacy regards transgender patients with a desire to achieve pregnancy [30].

Monjean et al. showed that vitrifying slow kinetics embryos are a procedure worth envisaging as it generated a non-negligible chance of pregnancy in a five-year prospective study on individually cryopreserved (Cryotop^®^) and warmed (Kitazato vitrification/warming kit) embryos with endpoints of transfer, implantation, and pregnancy rates. The pregnancy versus transfer was as high as 30.3% in the grade-three embryos based on development [31].

#### 3.2.3. Ovarian Tissue Banking

Freezing of ovarian tissue is another method used for medical or non-medical fertility preservation. For example, it allows retrieval of endocrine function, offers the possibility of natural conception after tissue transplantation, and postpones menopausal symptoms and related conditions [3,32].

Barriers to using ovarian tissue freezing are the need for surgical intervention to remove and replace the tissue, worries about the effects of removal of one ovary, and the risk after tissue transplantation of a possible IVF/ICSI procedure [33]. In contrast to a high success rate, the usage rate in ovarian tissue cryopreservation ranges around 8.7%, with a 57% live birth rate [34].

#### 3.2.4. Optimal Timing of Cryopreservation

Women’s age at the time of storage and the number of mature oocytes retrieved are predictor factors for future live births [1,3].

The optimal timing for a woman to freeze her eggs, with a much higher success rate, is under 35 years, as after this age, the quality and number of eggs decline [2,35]. Cryopreserving eggs at an earlier age can minimize the number of cycles necessary to obtain sufficient eggs and maximize egg quality, with the risk of never using them. With increasing age in women at the time of oocyte cryopreservation, outcomes are more negative. Women who are older at the time of ovarian stimulation for oocyte preservation require higher gonadotropin doses and storage of more oocytes of lower quality [36,37].

Still, an agreement is lacking regarding the optimal time of oocyte cryopreservation. Studies based on surveys reported ages between 36 and 38 at the moment of cryopreservation [1]; conversely, Mesen et al. found maximum benefits when cryopreservation is performed between 32 and 37 years, with little benefit at ages 25–30 [38].

#### 3.2.5. The Optimal Number of Oocyte

The number of oocytes retrieved should be individualized depending on the patient’s age, clinical circumstances, and ovarian reserve. Establishing the ovarian reserve using FSH and AMH values and the number of antral follicles can predict the oocyte numbers that can be retrieved [3,19].

Multiple cycles of controlled ovarian stimulation might be needed to obtain a sufficient number of oocytes [1]. Women can attempt a maximum of four oocyte retrieval cycles; the ideal number of retrieved oocytes is 20 [5,37].

Studies about fertility preservation in women demonstrated the high efficacity of this technology, as the probability of live birth increases with the number of cryopreserved oocytes, depending on the age of the oocyte. Existing data revealed a greater than 90% cumulative live birth rate (CLBR) using 24 frozen eggs and an 85.2% CLBR when 10–15 oocytes are used from a woman who cryopreserved her oocytes at 35 years or younger [1,39]. Storage of ten oocytes provides a probability of live births per thawed oocyte of 60.5% in women under 35 years compared to 29.7% in women aged over 35 years [40]. Cobo et al. [28] found that pregnancy rates are age-dependent and estimated that a minimum of 8–10 oocytes are necessary to achieve pregnancy, whereas Doyle et al. revealed that the use of 20 oocytes results in a CLBR of 60–80% in women between 35 and 38 years old [36].

A mathematical model can be used to predict the live birth-rate probability based on the number of cryopreserved oocytes and the woman’s age at the moment of cryostorage. For example, to obtain a 75% CLBR, women at 34, 37, or 42 years need to cryopreserve 10, 20, and 61 eggs, respectively [41].

A total of 15 to 20 oocytes retrieved in freeze-all cycles ensures a balance between the procedure’s safety and efficacy with individualized stimulation protocols [19].

Cryopreservation of a small number of oocytes can limit the success rate, whereas an excessive number of oocytes can result in unjustified costs and decrease the cost-effectiveness of additional recovery procedures.

#### 3.2.6. The Quality of Oocytes

Artificial intelligence (AI) was recently used to assess the quality of embryos and oocytes to create a highly predictable model for ART, which was able to recognize and classify embryos and oocytes [42] automatically.

In 1997, Kaufmann et al. created the first artificial neural network (ANN) to predict the success of a pregnancy, including the following parameters: the patient’s age, the number of competent oocytes, the number of transferred embryos, and the type of embryos (fresh or frozen) [43].

The development of AI has allowed the integration of new technologies in ART automation by processing oocytes (denudation by removing cumulus cells and oocytes positioning) to improve the efficiency and quality of ART [44].

Thus, AI for the selection [45] and the evaluation [46] of oocytes is a cheap, non-invasive method that is easy to integrate into the flow of ART. Cavalera et al. identified competent or incompetent oocytes with an accuracy of 91.03% [47]. Faramarzi et al. [46] used AI methods to quantify and evaluate oocyte development using a time-lapse technique, whereas Yanez et al. reported that a lack of embryonic viability is associated with certain gene expression transcriptomes of oocyte maturation [48].

**Figure 5:**
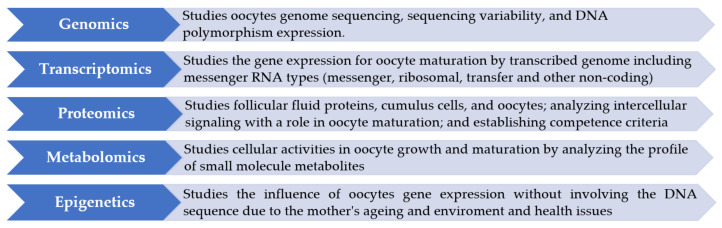
The OMICS technologies used for oocytes assessment.

The application of OMICS platform technologies in the field of egg freezing was found to be a challenging method of assessing the quality of oocytes [49,50] (Figure 5).

### 3.3. Safety of Freezing

The longer a person waits before becoming pregnant, the more likely it is that some diseases, accidents, or life circumstances will affect fertility or increase the risk of fetal abnormalities.

Recent studies demonstrated the safety of using vitrified oocytes on neonatal outcomes and genetic diseases, with the risk of congenital anomalies being the same as natural conception or following IVF treatment with fresh oocytes. No risks specific to freezing are known [51,52,53]. The risk of fetal loss and aneuploidies associated with age can be reduced using younger oocytes [54].

Chian et al. analyzed 165 pregnancies and 200 newborns and revealed that the incidence of congenital anomalies (2.5%) and the mean weight at birth are similar in infants born from cryopreserved oocytes to those spontaneously conceived. No obstetric and perinatal-specific risks to freezing are known [55]. The emotional health of children born to older mothers should be further studied and the associated child health outcomes [3,5].

The medical risks associated with cryopreservation are linked to (a) ovarian stimulation and (b) oocyte retrieval.

Ovarian hyperstimulation syndrome (OHSS) is the most common adverse event experienced with ovarian stimulation. OHSS can have mild, moderate, severe, or critical clinical manifestations. Mild and moderate OHSS appears in 3–6% of cases with headaches, fatigue, nausea, irritability, breast tenderness, abdominal pain, weight gain, and enlarged ovaries. Severe or critical OHSS occurs in 1–3% of stimulation protocols, are characterized by ascites and pleural effusion, shortness of breath, dehydration, vomiting, oliguria, hemoconcentration, thromboembolic events, and massive ovary enlargement, which are potentially life-threatening. Ovarian stimulation protocols with gonadotrophin-releasing hormone agonists to trigger ovulation reduce the risk of OHSS [1,16].

Oocyte retrieval procedures might result in pelvic pain, intraperitoneal bleeding, pelvic infection, damage to organs, ovarian torsion, and anesthesia risks [1,3,52]. In addition, the risk of bleeding is higher after more than 30 oocytes are retrieved [19].

IVF is also associated with risks for women who use their frozen-thawed oocytes to achieve a pregnancy. Multiple pregnancies related to age at storage and cerebral palsy sequelae due to premature birth are the most important risks associated with assisted reproduction techniques (ART). The risks also include pregnancy-related hypertension, operative delivery, and low birth weight [3,16].

Advanced maternal age is associated with a high risk of ectopic pregnancy, first-trimester losses, and obstetric risks such as preeclampsia, gestational diabetes, preterm birth, low birth weight, and cesarean birth [1,3,6]. These risks are the same in older women who achieved pregnancy using vitrified oocytes or IVF [51,54].

Long-term liquid nitrogen cryopreservation does not affect the euploidy rate or IVF success [1,53]. Therefore, women should be counseled regarding the procedure’s success rate and comprehend that it is not insurance against age-related infertility. However, with the new Israeli legislation, cryopreserved eggs can be fertilized and implanted until 54 years in this country [5].

### 3.4. The Usage Rate of Frozen Oocytes

The usage rate, a critical issue for elective egg freezing, is the percentage of women who use their frozen oocytes. Stoop et al. found that 50.8% of women reported the intention to use cryopreserved oocytes at some point in life; at three years after freezing time, 29.2% reported intention to use them. Hodes-Wertz et al. found that only 6% of women (11 of 183) used their oocytes, and only three conceived a child [56]. A survey of 23 women revealed that two used their frozen eggs, and one became pregnant [57].

In a retrospective observational study on 1468 women who underwent elective fertility preservation, published in 2016, Cobo et al. found that 9.3% of women used their oocytes at 39.2 years old, 2.1 years after freezing time, on average [40]. In another retrospective study of 6362 women that vitrified their oocytes for elective or oncofertility indications, Cobo et al. reported that the usage rate is 12.1% after 2.1 years of storage, with a mean age at the freezing time of 37.6 ± 3.5 years, and a mean age at the return of 39.9 ± 7 years [39].

Hammarberg et al. analyzed the reasons for the low usage rate of frozen oocytes, and the most common were found to be single-parent issues, the preference to conceive naturally, and not wanting to use a sperm donor [58], as well as insufficient knowledge and interest of women in the procedure at the optimal age range to perform FP (28–35 years).

Frozen oocytes can be stored for unlimited time without deterioration. However, legislation from the UK allows storage for a maximum of 10 years; after that, eggs must be discarded, limiting the success rate [3].

Decision regret is an indicator of the quality of health decisions. It involves negative emotions following a decision. In a retrospective survey of 201 women who electively cryopreserved their oocytes between 2012 and 2016, Greenwood et al. analyzed decision regret using the decision regret scale (DRS). Of the women interviewed, 51% had no decision regret, 33% reported mild decision regret, and 16% experienced moderate-severe decision regret, which indicates a low, but non-negligible, the prevalence of regret (mean of DRS was 10, with a range of 0–90). Factors associated with increased decision regret were a low number of frozen oocytes, a poor understanding of information and emotional support during the freezing process, and a reduced estimated probability of achieving a live birth using cryopreserved oocytes [59].

### 3.5. Ethical Considerations

The Ethics Committee of the American Society of Reproductive Medicine found that planned oocyte cryopreservation ethically allows increasing women’s reproductive autonomy and promotes social equality [60]. The first birth using vitrified human oocytes in the U.S. was reported in 2013 [61]; oocyte vitrification was allowed in the French Bioethics law of 2011 but is still debated [62,63,64]. The National Bioethics Council in Israel recommends OC to decrease age-related fertility [65], whereas in EU countries such as Austria, egg freezing for social reasons is currently not allowed but debated [66].

The ethical debate of the topic arouses many issues such as commercial exploitation, the medicalization of reproduction, women’s autonomy, idealization about the right time for a pregnancy, the impact of egg freezing on sex inequality, and professional norms [3,67,68].

Ethical perspectives involve evaluating the benefits, risks, costs, long-term implications, and proper counseling of those interested in elective fertility preservation of future efficiency and safety of future use [3,68].

Ethical arguments in favor of elective egg freezing focus on benefits to women and gender equality. Many women perceive egg freezing as an opportunity to stop the biological reproductive clock, acting as an insurance policy against age-related infertility, offering them reproductive autonomy and a chance to produce genetic children. Additionally, egg freezing at a younger age can reduce the risk of genetic abnormalities in children, which increases with the mother’s age [54,67].

Ethical arguments against the idea of fertility preservation for non-medical indication include the false sense of security of a future pregnancy produced by cryopreserved oocytes, which might support women in delaying childbearing, with secondary risks of late pregnancy for mother and child, and effects on the psychosocial development of the child due to late parenthood. In addition, many women who choose egg freezing for social reasons never return to use them is an argument against this procedure [3,68].

### 3.6. Cost-Effectiveness

Oocyte cryopreservation costs for age-related fertility loss may increase social inequality as it is only available to women who can afford the significant financial outlay [2].

State insurance systems do not stipulate elective egg freezing; this service is provided in the private sector and is paid for by the patients. However, some patients with private medical insurance may have part of the costs covered [5]. In 2012, Mertes et al. argued that transfer cycle costs should be covered, as with IVF cases, as should the costs of ovarian stimulation and oocyte retrieval and storage [69].

Companies offering to pay female employees to cryopreserve their oocytes raises ethical issues such as coercion and manipulation, making women feel that they need to delay motherhood to demonstrate dedication to their work. Young women view this act positively, offering them the chance to develop and reach the financial stability required for raising a child [2,16,67].

Cost-effectiveness defines procedure-related benefits. Elective egg freezing at a younger age can reduce the costs associated with infertility treatment and would be more successful and cost-effective [70] if performed at 35 [38] or 37 years of age [71]. An important cost consideration to discuss is the number of desired children; as the number of desired children increases, the procedure may be more cost-effective at a younger age [70].

When women decide to choose elective fertility preservation, to understand the costs involved in freezing (consultations, medications, blood tests, oocyte retrieval, freezing, and storage), IVF, and the usage rate, when estimating cost-effectiveness, women must consider the usage rate of the oocytes [1,10,16].

Loendersloot et al. demonstrated that the oocyte-freezing procedure is cost-effective if 61% of the women return to use their frozen eggs later in life [71]. In 2012, the American Society for Reproductive Medicine reported the slowing of the growing demand of FP procedures for delayed pregnancy in healthy women as oocyte cryopreservation or ovarian tissue cryopreservation (OTC) appears to be cost-effective [72].

## 4. Discussion

Oocyte cryopreservation is an elective method for fertility preservation against age-related infertility and involves ovarian hormonal stimulation, oocyte retrieval, freezing, and oocyte storage. Table 1 provides a synopsis of the main studies related to elective egg freezing.

In April 1986, *The Lancet Journal* reported the first human birth from a frozen oocyte, achieved in Australia. However, the use of the initial slow-freezing technique led to low oocyte survival, embryo development, and low pregnancy rates [17,51]. Over the past few years, oocyte cryopreservation has been challenging and has advanced considerably. 

Currently, special programs are available that help us calculate the number of oocytes needed to be harvested depending on the patient’s age to increase the risk of getting pregnant [41].

Through exhaustive analysis of the studies, we tried to synthesize some common points on patient’s attitudes towards oocyte preservation in all cases. A positive attitude on the issue was observed to vary between 17.8% and 100%, a higher level of education between 13.6% and 100%, concern about costs between 48.4% and 85.6%, and the status of the patient (single) was between 13.1% and 86%. This variability attests to the significant differences in the selection of cases, the period in which the follow-up of cases was performed, the distribution of patients in groups, and the criteria that were considered when developing questionnaires.

In the future and in the absence of international guidelines to support the standardization of IVF cryopreservation policy, many advanced techniques may be used (vitrification protocol, cryoprotective solutions, large tissue transplantation/whole ovary, biocompatible transplantable ovary, and artificial ovary) [52,82,83]. However, an ovarian autograft cannot be applied to leukemia or steroid-related cancers patients due to the increased risk of ovarian metastasis [83].

Vitrification, a novel ultra-rapid egg-freezing technique, was introduced, improving survival rates and clinical outcomes similar to those of fresh oocytes usage with a 90–97% survival rate, 71–79% fertilization rate, 17–41% implantation rate, and 4.5–12% clinical pregnancy rate per vitrified oocyte [3,5,52]. In addition, Gallardo et al. reported a vitrification method based on a dehydration protocol structured over two minutes, with evidence of cell survival after heating and the resumption of the intracellular process of cytokinesis, which improves the workflow of fertilization treatments in vitro [84].

To further achieve a standardized heating protocol, the survival rate of frozen embryos depends on establishing a precise concentration of extracellular cryoprotectants in the thawing/heating processes [85].

As of 2013, oocyte cryopreservation was no longer considered an experimental procedure by the American Society for Reproductive Medicine (ASRM). However, in 2012, the American Society for Reproductive Medicine recommended slowing the growing demand of FP procedures for delayed pregnancy in healthy women due to the procedure not being cost-effective. Loendersloot et al. stated that the procedure is cost-effective when 61% of the women return to use their frozen eggs later in life [71].

Since 2020, the ovarian tissue banking system (OTB) was no longer considered an experimental procedure. This technique involves removing a healthy ovary and cryopreserving it. The ovary is available for reimplantation after remission of disease, with most women regaining ovarian function. However, the procedure is used to maintain fertility in cancer patients and is not commonly available for non-medical indications [86].

Embryo cryopreservation has entered the discussion connected to IVF procedures, as specific amounts of embryos are not used, being considered surplus or abandoned post-IVF procedures. With this procedure, couples are confused about the ethical issues regarding the embryo, regarding it as a living entity, a baby, or as tissue. Gamete or EC and surrogacy are particular regarding transgender patients wanting to achieve pregnancy in certain circumstances.

The optimal age at social freezing is under 35 years (between 32 and 47 years). Cryopreserving eggs at an earlier age can minimize the number of cycles necessary to obtain sufficient eggs and maximize egg quality; older women require higher gonadotropin doses and storage of more oocytes of lower quality. Cryopreserved eggs can be fertilized and implanted until the age of 54 years in Israel.

The optimal number of oocytes is 20, with a maximum of four oocyte retrieval cycles. Storage of ten oocytes produces a probability of live birth per thawed oocyte of 60.5% in women under 35 years, compared to 29.7% in women aged over 35 years [40]. Thus, a mathematical model can be used to predict the live birth rate probability based on the number of cryopreserved oocytes and the woman’s age at the moment of cryostorage.

No risks specific to freezing are known [51,52,53]. The age-associated risk of fetal loss and aneuploidy can be reduced using younger oocytes [54]. Medical risks associated with cryopreservation surround those surrounding ovarian stimulation and oocyte retrieval.

The usage rate (the percentage of women who return to use their frozen oocytes) was 50.8% all-time and 29.2% at three years after the freezing moment.

Decision regret is an indicator of the quality of health decisions, measured by the decision-regret scale (DRS), consisting of negative emotions following the decision. Decision regret was absent in 51% of women who electively cryopreserved their oocytes (from 2012 to 2016).

However, various ethical considerations surround elective fertility preservation. Although it is still debated in several countries (Austria and France), it is recommended (Israel). Oocyte cryopreservation for age-related fertility loss is provided in the private sector, so costs are borne by the patients; some patients with private medical insurance may have part of the costs covered [5]. When estimating cost-effectiveness, women must consider the usage rate of the oocytes [1,10,16].

In this study, we only approached the female side of gamete conservation. The social freezing of sperm is different from the social freezing of oocytes, being less focused on preserving fertility, instead of reducing increased genetic risks due to advanced paternal age. Sperm freezing is most often recommended for male cancer patients, those with vasectomy, or those exposed to toxic environmental factors [87].

The future is represented by the involvement of nanotechnology, microfluidic biophysics, and oocyte culture using immature eggs from fresh ovarian tissue to establish the effectiveness of complete in vitro growth (IVG) of follicles, in vitro maturation (IVM), and in vitro fertilization technology (IVF) [83].

## 5. Conclusions

The social freezing topic is highly debated. Oocyte cryopreservation offers women the chance to have genetic children later in life, reducing uncertainty and anxiety. Socio-economic evolution is the most-noted reason influencing this new medical trend.

Fertility preservation clinicians should properly inform women about the safety, efficacy, benefits, usage rate, risks, and costs of the procedure and offer them real expectations to mitigate potential harms. Due to the novelty of the procedure, many women are less aware of the safety and efficacy of the procedure, including the possible complications of a pregnancy at an older age. Future research on usage rate, live birth rate, pregnancy outcomes, and long-term follow-up of children conceived using frozen-thawed oocytes are necessary.

### Limitations

Within the study, we selected CTs and RCTs from the PubMed and Web of Science Core Collection databases, searched by title and abstract topic; we did not analyze papers present in other databases.

## Figures and Tables

**Table 1:** Synopsis of studies according to patients’ perspectives and counseling assessment about elective egg freezing.

Author					Perspectives/Counselling							
	Year	Cases	Study Design	Age (Years)	Positive Attitude	Single	Higher Education	Costs Concerns	Number of OC	Birth	Usage Rate	Willing to Donate Eggs
Hodes-Wertz [56]	2013	183	Survey	23–46 38 ± 2.7	100%	84%	-	-	6–10	3	6%	63%
Daniluk [73]	2015	500	Self-report questionnaire	18–38	66%	58.2%	73%	85.6%	-	-	-	57.2–67%
Lewis [6]	2016	1064	Online questionnaire	18–65	17.8%	63.2%	-	-	-	-	-	-
Hammarberg [26]	2016	95	Survey	37.1 *	-	86%	89%	-	14.2	3	6%	-
Milman [74]	2017	1000	Cross-sectional	34.5 * (28–38)	-	23%	13.6%	-	-	-	-	-
Santo [75]	2017	444	Survey	33.2 ± 6.6	85.4%	13.1%	28.6%	49.3%	-	-	-	-
Inhorn [76]	2018	150	Audio-recorded interview	36.2–36.6	-	85%	100%	-	-	1	6%	-
Esfandiari [77]	2019	103	Survey	26–30	44.7%	23.3%	100%	-	-	-	-	57.2%
Tozzo [78]	2019	930	Survey	18–35	19.5%	48.7%	100%	48.4%	-	-	-	42.5%
Cardozo [79]	2020	171	Survey	21–45	-	50%	100%	59%	-	-	-	-
Johnston [80]	2020	656	Survey	18–60	>65%	27.6%	65.1%	-	-	-	-	-
Wafi [27]	2020	138	Online questionnaire	35.7 ± 0.9	-	83%	15%	-	17.6	13	20.3%	76.5%
Caughey [81]	2021	234	Online questionnaire	25–43	-	22.2%	36.8%	-	-	-	-	-

*, mean.

## Abbreviations

ART	Assisted Reproduction Techniques
AMH	Anti-Müllerian hormone
ASRM	American Society for Reproductive Medicine
CCS	Clinical Case Series
CLBR	Cumulative Live Birth Rate
DRS	Decision Regret Scale
IVF	In Vitro Fertilization
MA	Meta-Analysis
OHSS	Ovarian Hyperstimulation Syndrome
PGT-A	Preimplantation Genetic Tests For Aneuploidy
PRISMA	Preferred Reporting Items for Systematic Review and Meta-Analysis
CT	Clinical Trial
RCT	Randomized Clinical Trial
SEF	Social Egg Freezing
SR	Systematic Review

## References

[1] Alteri A., Pisaturo V., Nogueira D., D’Angelo A. (2019). Elective Egg Freezing without Medical Indications. Acta Obstet. Gynecol. Scand..

[2] ESHRE (2020). Female Fertility Preservation Guideline of the European Society of Human Reproduction and Embriology. https://www.eshre.eu/Guidelines-and-Legal/Guidelines/Female-fertility-preservation.

[3] Anderson R.A., Davies M.C., Lavery S.A., Royal College of Obstetricians and Gynaecologists (2020). Elective Egg Freezing for Non-Medical Reasons: Scientific Impact Paper No. 63. BJOG Int. J. Obstet. Gynaecol..

[4] Nasab S., Ulin L., Nkele C., Shah J., Abdallah M.E., Sibai B.M. (2020). Elective Egg Freezing: What Is the Vision of Women around the Globe?. Future Sci. OA.

[5] Shkedi-Rafid S., Hashiloni-Dolev Y. (2011). Egg Freezing for Age-Related Fertility Decline: Preventive Medicine or a Further Medicalization of Reproduction? Analyzing the New Israeli Policy. Fertil. Steril..

[6] Lewis E.I., Missmer S.A., Farland L.V., Ginsburg E.S. (2016). Public Support in the United States for Elective Oocyte Cryopreservation. Fertil. Steril..

[7] American College of Obstetricians and Gynecologists Committee on Gynecologic Practice and Practice Committee (2014). Female Age-Related Fertility Decline. Committee Opinion No. 589. Fertil. Steril..

[8] Bachmann G., MacArthur T.A., Khanuja K. (2018). Need for Comprehensive Counseling in Women Requesting Oocyte Cryopreservation. J. Womens Health 2002.

[9] Practice Committee of Society for Assisted Reproductive Technology, Practice Committee of American Society for Reproductive Medicine (2008). Essential Elements of Informed Consent for Elective Oocyte Cryopreservation: A Practice Committee Opinion. Fertil. Steril..

[10] Ben-Rafel Z. (2018). The Dilemma of Social Oocyte Freezing: Usage Rate Is Too Low to Make It Cost-Effective. Reprod. Biomed. Online.

[11] (2019). International Federation of Fertility Societies’ Surveillance (IFFS) 2019: Global Trends in Reproductive Policy and Practice, 8th Edition. Glob. Reprod. Health.

[12] Singer P., Wells D. (1983). In Vitro Fertilisation: The Major Issues. J. Med. Ethics.

[13] Potdar N., Gelbaya T.A., Nardo L.G. (2014). Oocyte Vitrification in the 21st Century and Post-Warming Fertility Outcomes: A Systematic Review and Meta-Analysis. Reprod. Biomed. Online.

[14] Garcia-Velasco J.A., Domingo J., Cobo A., Martínez M., Carmona L., Pellicer A. (2013). Five Years’ Experience Using Oocyte Vitrification to Preserve Fertility for Medical and Nonmedical Indications. Fertil. Steril..

[15] Takahashi N., Harada M., Oi N., Izumi G., Momozawa K., Matsuzawa A., Osuga Y. (2020). Preclinical validation of the new vitrification device possessing a feature of absorbing excess vitrification solution for the cryopreservation of human embryos. J. Obstet. Gynaecol. Res..

[16] Petropanagos A., Cattapan A., Baylis F., Leader A. (2015). Social Egg Freezing: Risk, Benefits and Other Considerations. Can. Med. Assoc. J..

[17] Argyle C.E., Harper J.C., Davies M.C. (2016). Oocyte Cryopreservation: Where Are We Now?. Hum. Reprod. Update.

[18] Lew R., Foo J., Kroon B., Boothroyd C., Chapman M., Australasian CREI Consensus Expert Panel on Trial evidence (ACCEPT) group (2019). ANZSREI Consensus Statement on Elective Oocyte Cryopreservation. Aust. N. Z. J. Obstet. Gynaecol..

[19] Mizrachi Y., Horowitz E., Farhi J., Raziel A., Weissman A. (2020). Ovarian Stimulation for Freeze-All IVF Cycles: A Systematic Review. Hum. Reprod. Update.

[20] Milachich T., Shterev A. (2016). Are There Optimal Numbers of Oocytes, Spermatozoa and Embryos in Assisted Reproduction?. JBRA Assist. Reprod..

[21] Barash O.O., Hinckley M.D., Rosenbluth E.M., Ivani K.A., Weckstein L.N. (2017). High Gonadotropin Dosage Does Not Affect Euploidy and Pregnancy Rates in IVF PGS Cycles with Single Embryo Transfer. Hum. Reprod. Oxf. Engl..

[22] Sekhon L., Shaia K., Santistevan A., Cohn K.H., Lee J.A., Beim P.Y., Copperman A.B. (2017). The Cumulative Dose of Gonadotropins Used for Controlled Ovarian Stimulation Does Not Influence the Odds of Embryonic Aneuploidy in Patients with Normal Ovarian Response. J. Assist. Reprod. Genet..

[23] Kuwayama M., Vajta G., Kato O., Leibo S.P. (2005). Highly Efficient Vitrification Method for Cryopreservation of Human Oocytes. Reprod. Biomed. Online.

[24] Seyhan A., Akin O.D., Ertaş S., Ata B., Yakin K., Urman B. (2021). A Survey of Women Who Cryopreserved Oocytes for Non-Medical Indications (Social Fertility Preservation). Reprod. Sci..

[25] Tan S.Q., Tan A.W.K., Lau M.S.K., Tan H.H., Nadarajah S. (2014). Social Oocyte Freezing: A Survey among Singaporean Female Medical Students. J. Obstet. Gynaecol. Res..

[26] Hammarberg K., Kirkman M., Pritchard N., Hickey M., Peate M., McBain J., Agresta F., Bayly C., Fisher J. (2017). Reproductive Experiences of Women Who Cryopreserved Oocytes for Non-Medical Reasons. Hum. Reprod..

[27] Wafi A., Nekkebroeck J., Blockeel C., De Munck N., Tournaye H., De Vos M. (2020). A Follow-up Survey on the Reproductive Intentions and Experiences of Women Undergoing Planned Oocyte Cryopreservation. Reprod. Biomed. Online.

[28] Goswami M., Murdoch A.P., Haimes E. (2015). To Freeze or Not to Freeze Embryos: Clarity, Confusion and Conflict. Hum. Fertil..

[29] Cattapan A., Baylis F. (2015). Frozen in Perpetuity: “abandoned Embryos” in Canada. Reprod. Biomed. Soc..

[30] Adeleye A., Sauer M. (2017). Managing the Unique Medical and Reproductive Needs of the Transgender Population. J. Reprod. Med..

[31] Montjean D., Pauly V., Gervoise-Boyer M., Amar-Hoffet A., Geoffroy-Siraudin C., Boyer P. (2019). Is It Worth It to Cryopreserve Embryos with Blastulation Delay at Day 5?. Zygote.

[32] Andersen C.Y., Kristensen S.G. (2015). Novel Use of the Ovarian Follicular Pool to Postpone Menopause and Delay Osteoporosis. Reprod. Biomed. Online.

[33] Balkenende E.M.E., van Rooij F.B., van der Veen F., Goddijn M. (2020). Oocyte or Ovarian Tissue Banking: Decision-Making in Women Aged 35 Years or Older Facing Age-Related Fertility Decline. Reprod. Biomed. Online.

[34] Hoekman E.J., Louwe L.A., Rooijers M., van der Westerlaken L.A.J., Klijn N.F., Pilgram G.S.K., de Kroon C.D., Hilders C.G.J.M. (2020). Ovarian Tissue Cryopreservation: Low Usage Rates and High Live-Birth Rate after Transplantation. Acta Obstet. Gynecol. Scand..

[35] Tsafrir A., Haimov-Kochman R., Margalioth E.J., Eldar-Geva T., Gal M., Bdolah Y., Imbar T., Hurwitz A., Ben-Chetrit A., Goldberg D. (2015). Ovarian Stimulation for Oocyte Cryopreservation for Prevention of Age-Related Fertility Loss: One in Five Is a Low Responder. Gynecol. Endocrinol. Off. J. Int. Soc. Gynecol. Endocrinol..

[36] Doyle J.O., Richter K.S., Lim J., Stillman R.J., Graham J.R., Tucker M.J. (2016). Successful Elective and Medically Indicated Oocyte Vitrification and Warming for Autologous in Vitro Fertilization, with Predicted Birth Probabilities for Fertility Preservation According to Number of Cryopreserved Oocytes and Age at Retrieval. Fertil. Steril..

[37] Wennberg A.-L. (2020). Social Freezing of Oocytes: A Means to Take Control of Your Fertility. Upsala J. Med. Sci..

[38] Mesen T.B., Mersereau J.E., Kane J.B., Steiner A.Z. (2015). Optimal Timing for Elective Egg Freezing. Fertil. Steril..

[39] Cobo A., García-Velasco J., Domingo J., Pellicer A., Remohí J. (2018). Elective and Onco-Fertility Preservation: Factors Related to IVF Outcomes. Hum. Reprod..

[40] Cobo A., García-Velasco J.A., Coello A., Domingo J., Pellicer A., Remohí J. (2016). Oocyte Vitrification as an Efficient Option for Elective Fertility Preservation. Fertil. Steril..

[41] Goldman R.H., Racowsky C., Farland L.V., Munné S., Ribustello L., Fox J.H. (2017). Predicting the Likelihood of Live Birth for Elective Oocyte Cryopreservation: A Counseling Tool for Physicians and Patients. Hum. Reprod..

[42] Wang R., Pan W., Jin L., Li Y., Geng Y., Gao C., Chen G., Wang H., Ma D., Liao S. (2019). Artificial Intelligence in Reproductive Medicine. Reproduction.

[43] Kaufmann S.J., Eastaugh J.L., Snowden S., Smye S.W., Sharma V. (1997). The Application of Neural Networks in Predicting the Outcome of Fertilization. Hum. Reprod..

[44] Meseguer M., Kruhne U., Laursen S. (2012). Full in Vitro Fertilization Laboratory Mechanization: Toward Robotic Assisted Reproduction?. Fertil. Steril..

[45] Kort J., Behr B. (2017). Biomechanics and Developmental Potential of Oocytes and Embryos. Fertil. Steril..

[46] Faramarzi A., Khalili M.A., Omidi M. (2019). Morphometric Analysis of Human Oocytes Using Time Lapse: Does It Predict Embryo Developmental Outcomes?. Hum. Fertil..

[47] Cavalera F., Zanoni M., Merico V., Bui T.T.H., Belli M., Fassina L., Garagna S., Zuccotti M. (2018). A Neural Network-Based Identification of Developmentally Competent or Incompetent Mouse wn Oocytes. J. Vis. Exp. JoVE.

[48] Yanez L.Z., Han J., Behr B.B., Pera R.A.R., Camarillo D.B. (2016). Human Oocyte Developmental Potential Is Predicted by Mechanical Properties within Hours after Fertilization. Nat. Commun..

[49] Dell’Aquila M.E., Cho Y.S., Martino N.A., Uranio M.F., Rutigliano L., Hinrichs K. (2012). OMICS for the Identification of Biomarkers for Oocyte Competence, with Special Reference to the Mare as a Prospective Model for Human Reproductive Medicine.

[50] Egea R.R., Puchalt N.G., Escrivá M.M., Varghese A.C. (2014). OMICS: Current and Future Perspectives in Reproductive Medicine and Technology. J. Hum. Reprod. Sci..

[51] Gunnala V., Schattman G. (2017). Oocyte Vitrification for Elective Fertility Preservation: The Past, Present, and Future. Curr. Opin. Obstet. Gynecol..

[52] Rybak E.A., Lieman H.J. (2009). Egg Freezing, Procreative Liberty, and ICSI: The Double Standards Confronting Elective Self-Donation of Oocytes. Fertil. Steril..

[53] Cobo A., Serra V., Garrido N., Olmo I., Pellicer A., Remohí J. (2014). Obstetric and Perinatal Outcome of Babies Born from Vitrified Oocytes. Fertil. Steril..

[54] Goold I., Savulescu J. (2009). In Favour of Freezing Eggs for Non-Medical Reasons. Bioethics.

[55] Chian R.-C., Huang J.Y.J., Tan S.L., Lucena E., Saa A., Rojas A., Ruvalcaba Castellón L.A., García Amador M.I., Montoya Sarmiento J.E. (2008). Obstetric and Perinatal Outcome in 200 Infants Conceived from Vitrified Oocytes. Reprod. Biomed. Online.

[56] Hodes-Wertz B., Druckenmiller S., Smith M., Noyes N. (2013). What Do Reproductive-Age Women Who Undergo Oocyte Cryopreservation Think about the Process as a Means to Preserve Fertility?. Fertil. Steril..

[57] Baldwin K., Culley L., Hudson N., Mitchell H., Lavery S. (2015). Oocyte Cryopreservation for Social Reasons: Demographic Profile and Disposal Intentions of UK Users. Reprod. Biomed. Online.

[58] Platts S., Trigg B., Bracewell-Milnes T., Jones B.P., Saso S., Parikh J., Thum M.Y. (2021). Exploring women’s attitudes, knowledge, and intentions to use oocyte freezing for non-medical reasons: A systematic review. Acta Obstet. Gynecol. Scand..

[59] Greenwood E.A., Pasch L.A., Hastie J., Cedars M.I., Huddleston H.G. (2018). To Freeze or Not to Freeze: Decision Regret and Satisfaction Following Elective Oocyte Cryopreservation. Fertil. Steril..

[60] Ethics Committee of the American Society for Reproductive Medicine (2018). Planned Oocyte Cryopreservation for Women Seeking to Preserve Future Reproductive Potential: An Ethics Committee Opinion. Fertil. Steril..

[61] Hickman T.N., McKenzie L.J., Schlenker T., Pinasco M.A. (2013). First Successful Delivery in Texas Using Vitrified Human Oocytes: A Case Report. J. Reprod. Med..

[62] Belaisch-Allart J., Brzakowski M., Chouraqui A., Grefenstette I., Mayenga J.-M., Muller E., Belaid Y., Kulski O. (2013). Social egg freezing: Which problems?. Gynecol. Obstet. Fertil..

[63] Frigout L. (2019). Préservation Dite « Sociétale » de la Fertilité de la Femme. https://dumas.ccsd.cnrs.fr/dumas-03139017/document.

[64] Bénard J., Calvo J., Comtet M., Benoit A., Sifer C., Grynberg M. (2016). Fertility preservation in women of the childbearing age: Indications and strategies. J. Gynecol. Obstet. Biol. Reprod..

[65] Shkedi-Rafid S., Hashiloni-Dolev Y. (2012). Egg Freezing for Non-Medical Uses: The Lack of a Relational Approach to Autonomy in the New Israeli Policy and in Academic Discussion. J. Med. Ethics.

[66] Kostenzer J., de Bont A., van Exel J. (2021). Women’s Viewpoints on Egg Freezing in Austria: An Online Q-Methodology Study. BMC Med. Ethics.

[67] Harwood K.A. (2015). On the Ethics of Social Egg Freezing and Fertility Preservation for Nonmedical Reasons. Medicolegal Bioeth..

[68] Dondorp W.J., De Wert G.M.W.R. (2009). Fertility Preservation for Healthy Women: Ethical Aspects. Hum. Reprod..

[69] Mertes H., Pennings G. (2012). Elective Oocyte Cryopreservation: Who Should Pay?. Hum. Reprod..

[70] Fritz R., Jindal S. (2018). Reproductive Aging and Elective Fertility Preservation. J. Ovarian Res..

[71] Van Loendersloot L.L., Moolenaar L.M., Mol B.W.J., Repping S., van der Veen F., Goddijn M. (2011). Expanding Reproductive Lifespan: A Cost-Effectiveness Study on Oocyte Freezing. Hum. Reprod..

[72] Hirshfeld-Cytron J., Grobman W.A., Milad M.P. (2012). Fertility Preservation for Social Indications: A Cost-Based Decision Analysis. Fertil. Steril..

[73] Daniluk J.C., Koert E. (2016). Childless Women’s Beliefs and Knowledge about Oocyte Freezing for Social and Medical Reasons. Hum. Reprod..

[74] Milman L.W., Senapati S., Sammel M.D., Cameron K.D., Gracia C. (2017). Assessing Reproductive Choices of Women and the Likelihood of Oocyte Cryopreservation in the Era of Elective Oocyte Freezing. Fertil. Steril..

[75] Santo E.V.E., Dieamant F., Petersen C.G., Mauri A.L., Vagnini L.D., Renzi A., Zamara C., Oliveira J.B.A., Baruffi R.L.R., Franco J.G. (2017). Social Oocyte Cryopreservation: A Portrayal of Brazilian Women. JBRA Assist. Reprod..

[76] Inhorn M.C., Birenbaum-Carmeli D., Birger J., Westphal L.M., Doyle J., Gleicher N., Meirow D., Dirnfeld M., Seidman D., Kahane A. (2018). Elective Egg Freezing and Its Underlying Socio-Demography: A Binational Analysis with Global Implications. Reprod. Biol. Endocrinol. RBE.

[77] Esfandiari N., Litzky J., Sayler J., Zagadailov P., George K., DeMars L. (2019). Egg Freezing for Fertility Preservation and Family Planning: A Nationwide Survey of US Obstetrics and Gynecology Residents. Reprod. Biol. Endocrinol. RBE.

[78] Tozzo P., Fassina A., Nespeca P., Spigarolo G., Caenazzo L. (2019). Understanding Social Oocyte Freezing in Italy: A Scoping Survey on University Female Students’ Awareness and Attitudes. Life Sci. Soc. Policy.

[79] Cardozo E.R., Turocy J.M., James K.E., Freeman M.P., Toth T.L. (2020). Employee Benefit or Occupational Hazard? How Employer Coverage of Egg Freezing Impacts Reproductive Decisions of Graduate Students. FS Rep..

[80] Johnston M., Fuscaldo G., Richings N.M., Gwini S., Catt S. (2020). Cracked Open: Exploring Attitudes on Access to Egg Freezing. Sex. Reprod. Health Matters.

[81] Caughey L.E., White K.M. (2021). Psychosocial Determinants of Women’s Intentions and Willingness to Freeze Their Eggs. Fertil. Steril..

[82] Cho E., Kim Y.Y., Noh K., Ku S.-Y. (2019). A New Possibility in Fertility Preservation: The Artificial Ovary. J. Tissue Eng. Regen. Med..

[83] Bioengineering and Evaluation of Artificial Ovaries for Fertility Preservation. Ph.D. Thesis. https://phd.leeds.ac.uk/project/226-bioengineering-and-evaluation-of-artificial-ovaries-for-fertility-preservation.

[84] Gallardo M., Saenz J., Risco R. (2019). Human Oocytes and Zygotes Are Ready for Ultra-Fast Vitrification after 2 Minutes of Exposure to Standard CPA Solutions. Sci. Rep..

[85] Capodanno F., Daolio J., De Feo G., Falbo A., Morini D., Nicoli A., Braglia L., Villani M., La Sala G.B., Parmegiani L. (2019). A Monocentric Analysis of the Efficacy of Extracellular Cryoprotectants in Unfrozen Solutions for Cleavage Stage Embryos. Reprod. Biol. Endocrinol. RBE.

[86] Practice Committee of the American Society for Reproductive Medicine (2019). Fertility Preservation in Patients Undergoing Gonadotoxic Therapy or Gonadectomy: A Committee Opinion. Fertil. Steril..

[87] Pennings G., Couture V., Ombelet W. (2021). Social Sperm Freezing. Hum. Reprod..

